# Cognitive outcome and its neural correlates after cardiorespiratory arrest in childhood

**DOI:** 10.1111/desc.13501

**Published:** 2024-04-01

**Authors:** Sharon Geva, Aparna Hoskote, Maneet Saini, Christopher A. Clark, Tina Banks, W. K. Kling Chong, Torsten Baldeweg, Michelle de Haan, Faraneh Vargha‐Khadem

**Affiliations:** ^1^ Department of Developmental Neurosciences University College London Great Ormond Street Institute of Child Health London United Kingdom of Great Britain and Northern Ireland; ^2^ Heart and Lung Division Institute of Cardiovascular Science Great Ormond Street Hospital London United Kingdom of Great Britain and Northern Ireland; ^3^ Department of Radiology Great Ormond Street Hospital London United Kingdom of Great Britain and Northern Ireland; ^4^ Neuropsychology Service Great Ormond Street Hospital London United Kingdom of Great Britain and Northern Ireland

**Keywords:** hippocampus, language, memory, striatum, thalamus

## Abstract

Hypoxia‐ischaemia (HI) can result in structural brain abnormalities, which in turn can lead to behavioural deficits in various cognitive and motor domains, in both adult and paediatric populations. Cardiorespiratory arrest (CA) is a major cause of hypoxia‐ischaemia in adults, but it is relatively rare in infants and children. While the effects of adult CA on brain and cognition have been widely studied, to date, there are no studies examining the neurodevelopmental outcome of children who suffered CA early in life. Here, we studied the long‐term outcome of 28 children who suffered early CA (i.e., before age 16). They were compared to a group of control participants (*n* = 28) matched for age, sex and socio‐economic status. The patient group had impairments in the domains of memory, language and academic attainment (measured using standardised tests). Individual scores within the impaired range were most commonly found within the memory domain (79%), followed by academic attainment (50%), and language (36%). The patient group also had reduced whole brain grey matter volume, and reduced volume and fractional anisotropy of the white matter. In addition, lower performance on memory tests was correlated with bilaterally reduced volume of the hippocampi, thalami, and striatum, while lower attainment scores were correlated with bilateral reduction of fractional anisotropy in the superior cerebellar peduncle, the main output tract of the cerebellum. We conclude that patients who suffered early CA are at risk of developing specific cognitive deficits associated with structural brain abnormalities.

**Research Highlights:**

- Our data shed light on the long‐term outcome and associated neural mechanisms after paediatric hypoxia‐ischaemia as a result of cardiorespiratory arrest.
- Patients had impaired scores on memory, language and academic attainment.
- Memory impairments were associated with smaller hippocampi, thalami, and striatum.
- Lower academic attainment correlated with reduced fractional anisotropy of the superior cerebellar peduncle.

## 1 INTRODUCTION

Neonatal hypoxia‐ischaemia (HI) can have a circulatory or respiratory origin (Fatemi et al., 2009; Thompson, Omizzolo et al., 2014; van Schie et al., 2007; Young et al., 2015), and its adverse effects on brain structure and function are mediated through various molecular pathways (Fatemi et al., 2009). It is as yet unknown whether HI of different aetiological origins can result in different outcomes. However, the most common patterns of brain injury associated with HI in term neonates include damage to: (i) the hippocampus which underpins cognitive memory and learning, the basal ganglia (specifically the neostriatum; comprised of the caudate nucleus and the putamen) which support habit formation and skill learning, and the thalamus (Cowan et al., 2003); and (ii) the watershed zones, which usually affects the white matter, although, in more severely affected children, can also cause damage to the surrounding cortex (Fatemi et al., 2009).

These brain structures affected by early HI form part of various functional and structural neural circuits, and therefore, damage to these structures can result in behavioural sequelae in various developmental domains, including motor and cognitive functions. The developmental outcome can vary in severity, from an overt neurological outcome, such as cerebral palsy, through subtle cognitive abnormalities, to normal development (Fatemi et al., 2009). Adverse cognitive outcomes, especially affecting memory function, have been reported previously, after diverse aetiologies leading to neonatal hypoxia‐ischaemia (Gadian et al., 2000; Vargha‐Khadem et al., 1997). Subsequent cohort studies examined children with specific HI aetiologies, including children treated for acute hypoxemic respiratory failure (AHRF; Cooper et al., 2015), and children treated during the neonatal period for transposition of the great arteries (TGA) using a corrective arterial switch operation (Muñoz‐López et al., 2017). A substantial number of those patients (45% in Cooper et al., 2015; 33% in Muñoz‐López et al., 2017) showed marked memory impairments (defined by clinical criteria using standardised tests), and on the group level, this was associated with significant reduction in bilateral hippocampal volume, on a background of otherwise normal cognitive and neurological outcome.

Cardiorespiratory arrest (CA) is another well‐known cause of HI. The cognitive and motor function following CA in adults has been studied before (see below). However, CA is relatively rare in children as compared to adults (Engdahl et al., 2003; Holmberg et al., 2019; Tsao et al., 2022), and this may partly explain the scarcity of data on functional outcomes following CA in children. In addition, in children, cardiac arrest usually has different aetiology than in adults: the most common cause of paediatric cardiac arrest is hypoxia and rarely a primary cardiac event, unlike in adults where the common cause is ventricular fibrillation (Manole et al., 2009; Tress et al., 2010). Moreover, during infancy and childhood neuronal maturation and synaptogenesis still occur. As a consequence, the sequelae of childhood cardiac arrest and the recovery patterns could differ from those seen in adults (Tress et al., 2010). Put together, it is suggested that one cannot adequately infer about the outcome of developmental CA from studies of adult CA. In order to shed further light on outcome following paediatric CA, we therefore studied a rare cohort of children and adolescents who had suffered from CA, with the aim of understanding their long‐term cognitive outcome in relation to the integrity of known neuroanatomical targets of HI.

In paediatric populations, two large cohort studies examined general cognitive outcome 12 months after CA, defining children's impairment status according to standardised tests of The Mullen Scales of Early Learning for children below 6 years of age, or two subscales from the Wechsler Abbreviated Scale of Intelligence (WASI) for older children (Slomine et al., 2016, 2018a, 2018b). Most patients who had out‐of‐hospital CA (total *n* = 60) performed within the impaired range (Slomine et al., 2016), while among those who had in‐hospital CA (total *n* = 100), less than half showed similar impairment (Slomine et al., 2018a). Further testing using additional standardised tests of the older children (>6 years of age; *n* = 41), revealed that some of the patients had impairments in verbal recall (∼20%, tested with the California Verbal Learning Test), visual recall (∼50%, Rey–Osterrieth complex figure), verbal fluency (∼40%), and motor functioning (∼40%, Grooved Pegboard and Beery‐Buktenica Visual‐Motor Integration) (Slomine et al., 2018b). A single small study of cardiac arrest in adolescents found that six out of eight patients whose memory status could be assessed, showed mnemonic impairments early after the event. In a follow‐up examination (minimum 6 months post CA) two of these adolescents fully recovered, and the remaining four had residual deficits (Maryniak et al., 2008).

As data on outcome following early CA are scarce, below we briefly review the literature on cognitive outcome following adult CA. In adults, it is now well‐established that some patients who had cardiac arrest suffer mild (33%–66% of survivors) or severe (7%–28%) memory impairments (Alexander et al., 2011; Bunch et al., 2004; Cronberg et al., 2009; Grubb et al., 1996; Mateen et al., 2011; Moulaert et al., 2009; O'Reilly et al., 2003; Sauve, 1995; Sunnerhagen et al., 1996; Tiainen et al., 2007); (but see Bertini et al., 1990). In seminal studies of 56 adult cardiac arrest patients, Yonelinas and colleagues (Yonelinas et al., 2002, 2004) found that recall was significantly more affected than recognition, with the latter being supported by both recollection and familiarity, and the former only by recollection (Quamme et al., 2004). A review concluded that performance in all cognitive domains can be compromised (Moulaert et al., 2009), with executive functions most commonly affected (Cronberg et al., 2009; Drysdale et al., 2000; Lim et al., 2004; Nunes et al., 2003). Lim et al. (2004) found no patients with isolated memory impairment, arguing that following CA, memory impairments always co‐occur with other cognitive dysfunctions. When they occur, the memory impairments are chronic and affect recognition as well as recall, suggesting non‐focal brain injury (Drysdale et al., 2000; Grubb et al., 2000). However, verbal long term memory was found to be more affected than visual long term memory (Grubb et al., 1996). It is well‐documented that impairments of memory and other cognitive functions affect independence and quality of life of both patients and carers (Pusswald et al., 2000).

To summarise, studies have demonstrated that early HI of various aetiologies can result in both isolated and non‐isolated memory deficits. Data from children who suffered HI following CA are limited, but suggest that memory impairments persist alongside other cognitive and motor dysfunction. Similarly, studies of adult CA suggest that memory impairments are common, though rarely appear in isolation.

Here we studied the long‐term cognitive outcome of a cohort of children and adolescents who suffered a cardiorespiratory arrest early in life. We also aimed at defining the neural mechanisms underlying these impairments, as, to the best of our knowledge, this has not been documented in this population.

Based on the behavioural results from adult studies, and from studies of children who suffered acute episodes of hypoxia‐ischaemia following CA, as well as other aetiologies, we hypothesised that impairments would be found in the domains of memory (Cooper et al., 2015; Lim et al., 2004; Maryniak et al., 2008; Muñoz‐López et al., 2017, Slomine et al., 2018b), language (Slomine et al., 2018b) and general cognitive outcome (Lim et al., 2004; Slomine et al., 2016, 2018a, 2018b).

We attempted to replicate previous findings showing that older age at CA (Slomine et al., 2016, 2018a), longer duration of CA (Grubb et al., 1996; Ichord et al., 2018) and receiving Extracorporeal membrane oxygenation (ECMO) treatment are reliable predictors of lower cognitive performance (Ichord et al., 2018; Meert et al., 2018, 2016).^1^

We also examined whether the patient cohort had grey and white matter injury to structures commonly affected by HI (i.e., hippocampus, thalamus, dorsal striatum of the basal ganglia, and white matter tracts), and whether this damage is associated with specific domains of cognitive dysfunction. Specifically, based on previous studies of neurodevelopmental mechanisms of memory, language and academic function, we hypothesised that in the patient cohort, memory dysfunction will be associated with damage to the hippocampus and fornix (Cooper et al., 2015; Dogan et al., 2022; Mishkin et al., 1998; Muñoz‐López et al., 2017; Vargha‐Khadem et al., 1997), as well as the thalamus (Dzieciol et al., 2017; Sweeney‐Reed et al., 2021); language ability will be associated with the integrity of the basal ganglia (Martinez‐Biarge et al., 2010; Martinez‐Biarge, Bregant et al., 2012; Martinez‐Biarge, Diez‐Sebastian et al., 2012), arcuate fasciculus/superior longitudinal fasciculus (AF/SLF), and inferior longitudinal fasciculus/Inferior fronto‐occipital fascicle (ILF/IFOF) (Dick et al., 2014; Friederici, 2015); and academic attainments will be associated with integrity of the superior cerebellar peduncle (the major efferent pathway of the cerebellum which sends information to the cortex through the thalamus, Albazron et al., 2019).

## 2 METHODS

### 2.1 Participants

From a database of patients who suffered a substantial cardiorespiratory arrest (CA) and were seen in Great Ormond Street Hospital between the years 1997 and 2014, we initially included all those who would have been 8–22 years of age at the time of study participation. Inclusion criteria were: no history of other developmental or genetic disorders or conditions which may affect neurological function, living in the UK, term birth, both patient and parent/guardian are English‐speaking, and MRI compatibility. Out of 326 patients, 182 (55%) were deceased, and further 91 were excluded based on the above criteria. Among the 53 patients who were included, 25 did not participate in the study (13 refused; 11 had missing contact details; one agreed but did not attend). The remaining 28 patients participated in the study. For full recruitment details see Figure S1. For clinical and demographic details of each participating patient see Table S1. The following medical variables describe the patient cohort: age at CA (mean: 3.4 ± 4.4 years), total arrest time (mean 10.8 ± 19.8 min; coded as 1 min in 3 cases where medical notes stated ‘short downtime’), ECMO treatment (14 yes/14 no), number of days on ECMO (*n* = 14; mean: 9 ± 4.11), CA location (two out of hospital/27 in hospital), mechanical circulatory support with ventricular assist device (1 yes/27 no), and heart transplant (6 yes/22 no).

To evaluate the representativeness of the recruited sample, we compared demographic and clinical variables of the patients who participated in the study, to those of the 25 patients who met the inclusion criteria but did not participate. The two patient groups did not differ on any of the variables (see Table S2), suggesting that assessment of only half of the eligible patients did not bias our final sample with regard to medical and demographic variables.

Thirty healthy participants were recruited mainly from schools in the London area to act as a comparison control group. Inclusion criteria were age 8–22 years at the time of study participation, no relevant medical history, no developmental disorders, term birth, and both child and parent are English‐speaking. Inclusion was determined based on answers given by parents to a detailed medical questionnaire administered via telephone. Two healthy controls were later excluded due to abnormal findings on the MRI scan, resulting in a control group of 28 participants.

Socio‐Economic‐Status (SES) of participants was defined using the National Office of Statistics data (Income Deprivation Affecting Children Index; IDACI). Data were missing for two patients who live outside of England, and for one healthy participant whose post code was not obtained. Both groups were representative of the general population (one sample *t*‐test, *p* > 0.05 for both groups). The group of patients did not differ from the control group in age, sex, handedness (Oldfield, 1971), or SES (see Table 1). Group demographic and clinical information is provided in Table 1 and Table S2.

**TABLE 1:** Demographic information of the groups of patients and healthy controls.

Variables		Controls	Patients	Statistical test	p‐value
Age at first CA (years)	Mean (SD)	N/A	3.4 (4.4)	N/A	N/A
	Range		0 – 13.8		
Time between CA and test (years)	Mean (SD)	N/A	8.5 (3.9)	N/A	N/A
	Range		1 – 15		
Age at test (years)	Mean (SD)	12.4 (3.2)	12.4 (3.3)	t‐test	0.973
	Range	8 – 21	8 – 20		
Sex	F	18	17	Chi square	0.783
	M	10	11		
SES	Mean (SD)	16 (10.5)	17 (9.4)		
IDACI rank (k)	Range	1 – 32.3	0.6 – 30.3	t‐test	0.833
SES	Mean (SD)	5.6 (3.1)	5.8 (2.9)		
IDACI decile	Range	1 – 10	1 – 10	Mann‐Whitney	0.795
Handedness	Right	25	25	Chi square	0.717
	Left	1	2		
	Ambidextrous	2	1		

Abbreviations: CA, cardiac arrest; IDACI, income deprivation affecting children index; SES, socio‐economic‐status.

### 2.2 Neuropsychological assessment

All participants completed a series of standardised cognitive tests at The Wolfson Centre, UCL Great Ormond Street Institute of Child Health. For most participants assessments were completed over one day. Neuropsychological assessments took around 3.5 h in total, usually with a lunch break in the middle. Participants were given as many breaks as needed. The following cognitive tests were administered: (a) **Wechsler Abbreviated Scale of Intelligence** (WASI; Wechsler, 1999): measuring verbal IQ (VIQ; based on two subtests) and performance IQ (PIQ; based on two subtests), and generating a full‐scale IQ index score (FSIQ; based on the four subtests); (b) **The Children Memory Scale** (CMS; ages 8–16; Cohen, 1997) or the **Wechsler Memory Scale** (WMS; ages 16 and above; Wechsler, 2009): measuring immediate and delayed verbal and visual memory recall (generating a general memory index score, based on these four recall sub‐scales), and a verbal recognition memory score (based on recognition in two delayed verbal tests). Here we focus on delayed recall, in line with previous studies of memory impairments following HI in children and adults (Quamme et al., 2004; Vargha‐Khadem et al., 2001; Yonelinas et al., 2002, 2004); (c) **Rivermead Behavioural Memory Test** (RBMT; children version for ages 8–10; version II for ages 11 and above; Wilson et al., 1991): measuring everyday episodic memory; (d) **Wechsler Individual Achievement Test—II** (WIAT‐II; Smith, 2001): measuring academic attainments. Four subtests were used from this test battery: Numerical Operations, Word Reading, Reading Comprehension, and Spelling. (e) The **Expression, Reception and Recall of Narrative Instrument** (ERRNI; Bishop, 2004): measuring language ability using a narrative test, and generating five scores: (i) Ideas 1: amount of information given in initial storytelling (while looking at a picture book); (ii) Ideas 2: amount of information given in the second storytelling (after 30 min delay, without looking at the picture book); (iii) Forgetting level (between the two storytelling sessions); (iv) Mean Length of Utterance (MLU; averaged across both storytelling sessions); (v) Comprehension score: accuracy of answers given to a number of questions presented to the participant while looking at the pictures, at the end of the testing session. (f) The **Sunderland Memory Questionnaire for Children and Adolescents** (Sunderland et al., 1983) was completed by the participants’ primary carer.

### 2.3 Imaging data acquisition

MR images were acquired using a 1.5T Siemens Avanto (Germany) MRI scanner at Great Ormond Street Hospital, London. For most participants the MRI scan was performed on the same day as the neuropsychological assessments. The following scans were acquired: T1‐weighted Fast Low Angle Shot (FLASH) scan (repetition time (TR) = 11 ms, echo time (TE) = 4.94 ms, flip angle = 15°, field of view = 224 × 256 mm, 176 slices, sagittal plane, voxel size: 1 × 1 × 1 mm); T2‐weighted scan (TR = 4920 ms, TE = 101 ms, flip angle = 90°, field of view = 220 × 256 mm, 25 slices, voxel size: 7 × 6 × 4 mm); and a Diffusion Tensor Image (DTI; TR = 7300 ms, TE = 81 ms, field of view: 240 × 240 mm, 60 slices, axial plane, voxel size: 2.5 × 2.5 × 2.5 mm, 60 gradient directions, b‐value: 1000 s/mm^2^). Four patients did not have an MRI scan (one found to be non‐MRI compatible prior to scanning, one stopped the scan in the middle, for one the parent declined the scan following testing, and for one patient the MRI data were unusable because of artefacts created by braces).

One of the authors, a consultant paediatric neuroradiologist (WKKC) visually inspected all T1‐weighted and T2‐weighted MRI scans, while being blinded to participants’ diagnosis or group affiliation. See Table S3 for radiological findings of all the participants.

### 2.4 Imaging data pre‐processing

#### 2.4.1 Processing of T1‐weighted images

T1‐weighted FLASH images were processed using the Statistical Parametric Mapping software (SPM12; Wellcome Centre for Human Neuroimaging, London, UK; https://www.fil.ion.ucl.ac.uk/spm/) running in the Matlab environment (2021a Mathworks, Sherbon, MA, USA). Images were segmented into grey matter (GM), white matter (WM), cerebro‐spinal fluid (CSF) and other tissue type based on probability maps, using the Segment tool. This procedure combines tissue segmentation, bias correction, and spatial normalization in a single unified model (Ashburner & Friston, 2005). Normalisation was carried out using Diffeomorphic Anatomical Registration Through Exponentiated Lie Algebra (DARTEL, Ashburner, 2007), which creates templates and normalises individual scans to MNI space based on all participants’ images (*n* = 52). Images were smoothed using an 8 mm Full‐Width at Half Maximum (FWHM) Gaussian kernel. Tissue volumes were calculated using the Tissue Volume utility in SPM12, and intra‐cranial volume (ICV) was defined as a combination of whole brain GM, WM and CSF volumes.

#### 2.4.2 Processing of diffusion weighted images

**FIGURE 1:**
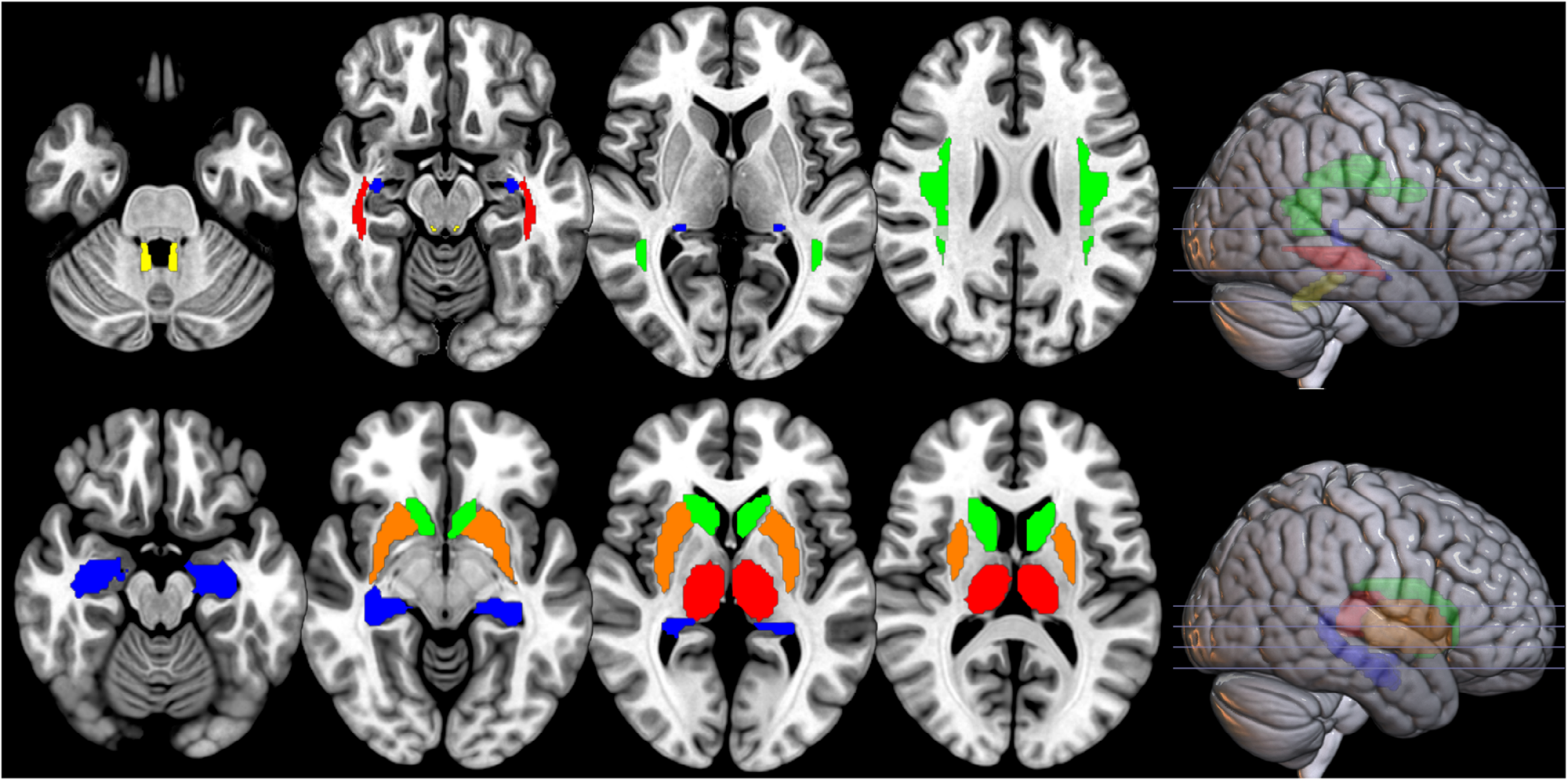
White matter tracts and grey matter structures of interest. Top panel: white matter tracts of interest (JHU Atlas; Ling and Rumpel, 2006): Yellow: superior cerebellar peduncle, Red: ILF/IFOF, Green: AF/SLF, Blue: Fornix. Bottom panel: grey matter regions of interest: Blue: hippocampus, Orange: putamen, Red: thalamus, Green: caudate nucleus. Structures are displayed on axial slices in MNI space.

Processing of DWI was carried out using the FMRIB's Software Library (FSL; http://www.fmrib.ox.ac.uk/fsl, Smith et al., 2004). First, eddy current distortions and head movements were corrected by registering all DTI volumes to the first b0 volume, using the eddy correct tool. Next, the FSL Brain Extraction Tool (BET) was applied to obtain a binary brain mask for each participant. A diffusion tensor model was fitted at each voxel using DTIFIT to obtain the eigenvalues (*λ*1, *λ*2, *λ*3) and eigenvectors (V1, V2, V3) of the diffusion tensor matrix. Maps of Fractional Anisotropy (FA), Mean Diffusivity [MD = (*λ*1 + *λ*2 + *λ*3)/3], and Radial Diffusivity [RD = (*λ*2 + *λ*3)/2] were generated based on these eigenvalues. Each dataset was visually inspected for data quality by someone who was blinded to group affiliation of the participants, and those with severe artefact were discarded from further analysis. The data from one patient and one healthy volunteer were discarded because of motion artefacts created by the participant's own movement. Four additional datasets (two patients and two healthy volunteers) were discarded due to motion artefacts related to table motion during data acquisition. We then applied nonlinear registration of each image into standard space provided by FSL (FMRIB58‐FA standard‐space which is an FA template generated by averaging 58 diffusion MRI data images, in MNI152 space). Lastly, a mean image was created based on data from the remaining participants (*n* = 46) and skeletonised for the whole group. It was thresholded with the default threshold value of 0.2. Masks were created for studying specific tracts of interest using the JHU Atlas (Ling & Rumpel, 2006), binarized, and mean FA, MD and RD values were extracted from the tracts of each participant. Tracts of interests are presented in Figure 1.

#### 2.4.3 Volumetry of subcortical structures

We performed automated segmentation of the subcortical structures (bilateral hippocampus, thalamus, caudate nucleus and putamen as regions of interest, brain stem as control region) using FSL‐FIRST v.6.0 (Patenaude et al., 2011) from all participants’ T1‐weighted MRIs in native space. Volumes were segmented in each hemisphere and visually inspected for accuracy. One control participant was excluded for poor registration and outlier values for ICV and all subcortical structures (outliers defined as values > Mean + 3SD of the control group). Volumetric measurements were normally distributed (Shapiro‐Wilk test, *p* > 0.05 for all). Regions of interests are presented in Figure 1.

### 2.5 Data analysis

All data analyses were carried out in IBM SPSS Statistics for Windows (version 27.0, IBM Corp., Armonk, New York, USA)

#### 2.5.1 Behavioural data analysis

For dimensionality reduction, we ran principal component analyses (PCAs) on the behavioural scores of the different tests (following Corbetta et al., 2015; Ramsey et al., 2017; Salvalaggio et al., 2020) for the entire sample (patients and control participants combined). We ran three independent PCAs based on a priori theoretical grouping of the administered subtests into three domains: (i) Memory (Sunderland, CMS/WMS verbal delayed score, RBMT and ERRNI Forgetting); CMS/WMS visual delayed scores were not included as previous studies (Alonso et al., 2023; Buck et al., 2021) suggested that this score is not sensitive enough to small variability in delayed memory abilities because memory load in this test is low; and in line with an adult study, showing that verbal long term memory was more affected than visual long term memory (Grubb et al., 1996); (ii) Language (ERRNI scores other than Forgetting); and, (iii) Attainment (WIAT subtests). In order to maximize the use of the data, we eliminated the WIAT reading comprehension scores from the final PCA, as 12 participants did not complete this subtest. However, the components derived from four versus three WIAT subtests were highly positively correlated (Pearson's *r* = 0.98, *p* < 0.001).

We used oblique rotation, because we assumed the components are not orthogonal. Components had to satisfy two criteria: (i) the eigenvalues had to be >1; (ii) the percentage of variance accounted for had to be >10%. The theoretically driven PCA approach taken here follows the recommendations of previous studies of clinical populations (Corbetta et al., 2015; Ramsey et al., 2017; Salvalaggio et al., 2020). Although we did not limit a priori the number of components to be extracted from each PCA, in each domain, we obtained only one component that satisfied the two criteria above, and consequently, accounted for the majority of the variance. Although our sample size was relatively small for PCA analysis, all criteria indicated adequacy of the method: Kaiser–Meyer–Olkin measure of adequacy was above 0.5, Bartlett's test of sphericity was significant, and factor loading values were all equal to, or higher than 0.3. See Table S4 for further details on the three PCAs.

To examine group differences (patients vs. controls) in behavioural scores we performed a 2 × 3 MANCOVA (two groups, three behavioural domains, age at test, sex and PIQ as covariates), and we report main effects and interaction effects. When main and/or interaction effects were significant, we followed the MANCOVA with post‐hoc independent sample 1‐tailed *t*‐tests. As behavioural data was partial for some participants, the MANCOVA includes only 24 patients and 24 control participants. We therefore also report *t*‐test results of the full sample. Behavioural data and group comparisons for all administered tests are reported in Tables S5A–C.

Within the patient group, we examined whether behavioural outcome was associated with cardiac arrest factors (age at CA and total arrest time; using 1‐tailed partial correlations controlling for age at test, and Benjamini–Hochberg FDR corrections for multiple comparisons) (Benjamini & Hochberg, 1995), or ECMO treatment (Mann‐Whitney *U* Test), and we report the associated *r* and *q*, or *U* and *p*, respectively.

#### 2.5.2 Group differences in tissue volumes and microstructure

To examine group differences (patients vs. controls) in tissue volumes and microstructure we performed a series of MANCOVAs, with age at test and sex as covariates, and we report main effects of group and interaction effects. MANCOVAs were followed by post‐hoc independent sample 1‐tailed *t*‐tests.

MANCOVAS were performed for the following (i) 2 × 3 MANCOVA of whole‐brain tissue volume: GM, WM and CSF; (ii) 2 × 3 MANCOVA of whole‐brain diffusion parameters: FA, MD and RD; (iii) 2 × 4 × 2 MANCOVA of subcortical grey matter structures of interest (four subcortical structures: hippocampus, thalamus, caudate nucleus, putamen; two hemispheres: left and right); (iv) 2 × 3 × 2 MANCOVA of white matter tracts of interest (three tracts: AF/SLF, ILF/IFOF, superior cerebellar peduncle (SCP); two hemispheres: left and right). We also compared the two groups for FA values of the fornix using independent sample t‐test (this was not included in the MANCOVA as TBSS does not distinguish right and left hemispheric portions of the fornix). When testing for group differences in specific brain structures we also report the influence of controlling for global tissue integrity, that is, whole brain grey matter volume for (iii) and global FA for (iv).

We then examined the association between global tissue parameters (GM and WM volume, and FA, RD and MD) and cardiac arrest factors (age at CA and total arrest time, using 1‐tailed partial correlations controlling for age at test, and Benjamini–Hochberg FDR corrections for multiple comparisons) (Benjamini & Hochberg, 1995), or ECMO treatment (Mann‐Whitney *U* Test), and we report the associated *r* and *q*.

#### 2.5.3 Structure–function relationships

We aimed to establish a relationship between brain abnormalities and cognitive performance, using partial correlations, controlling for age at testing, and Benjamini–Hochberg FDR corrections for multiple comparisons (Benjamini & Hochberg, 1995). We report the *r* and *q* of all correlations, separately for the patient and control groups.

We correlated the components obtained in the three PCAs of the behavioural performance (i.e., memory, language and academic attainment), with: (i) White matter abnormality: extracted mean FA for each participant in the fornix, and left and right AF/SLF, ILF/IFOF and SCP (Matejko & Ansari, 2015; Moeller et al., 2015); and, (ii) Grey matter abnormality: bilateral volumes of the hippocampus, thalamus, caudate nucleus and putamen. As a control analysis, we confirmed that behavioural performance did not correlate with (i) global FA; or (ii) global grey matter volume, even without adjusting for multiple comparisons (i.e., unadjusted *p* > 0.05).

## 3 RESULTS

### 3.1 Cognitive performance

A 2 × 3 MANCOVA of behavioural scores revealed a significant main effect of group (*F*_(1,43) _= 8.15, *p* = 0.007, partial eta squared = 0.159), but no significant effect of behavioural domain (*F*_(2,43) _= 1.25, *p* = 0.284, partial eta squared = 0.028), and no significant interaction between group and behavioural domain (*F*_(2,43) _= 0.63, *p* = 0.487, partial eta squared = 0.014).

**FIGURE 2:**
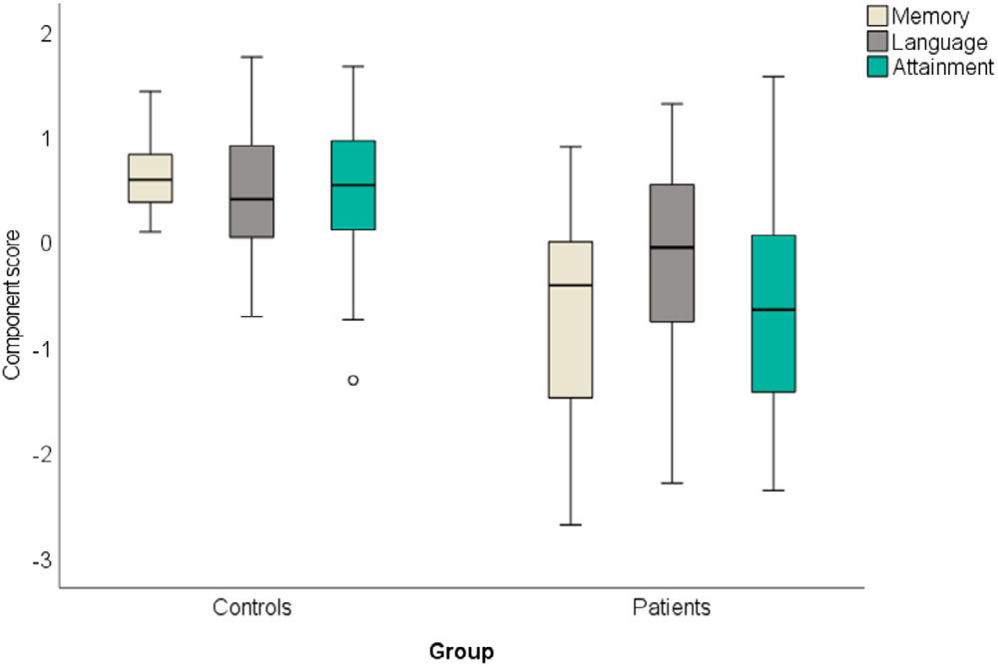
Group differences in cognitive outcome. Y axis represents the factor scores. The box represents the interquartile range (IQR), and the hinges represent the minimum and maximum values (excluding outliers). Small full circles represent values which are > 1.5 times the IQR, below Q1 or above Q3.

Independent sample *t*‐tests including the entire cohort showed that patients had significantly lower scores compared to the control group, on all three components (Memory: *t*_(29.01) _= 6.11, *p* < 0.001, Cohen's *d* = 0.75; Language: *t*_(41.9) _= 3.03, *p* = 0.004, Cohen's *d* = 0.94; Attainment: *t*_(49) _= 4.38, *p* < 0.001, Cohen's *d* = 0.86). See Figure 2. Similar results were obtained for the subgroup of participants included in the MANCOVA above (*n* = 48, see Section 2.5.1 Behavioural data analysis).

Age at cardiac arrest, its duration or ECMO treatment were not associated with behavioural scores (*q/p* > 0.05 for all). Table S5A provides group statistics and number of participants who performed within the impaired range, in each of the subtests. Table S5B presents comparison of behavioural scores across groups (patients vs. control group), and compares the patient group to the population mean (*μ*) on the standardised tests. We note that compared to standard population scores (where these scores are available), the patient group had significantly lower performance on most of the subtests included in the Memory component, and some of the subtests included in the Attainment component, but only in one subtest included in the Language component (see Table S5B). Table S5C (excel file) presents a colour code of each participant performance across all the standardised tests. Table S6A provides statistics of the correlation analyses.

### 3.2 Group differences in tissue volume and microstructure

#### 3.2.1 Whole brain measurements

**FIGURE 3:**
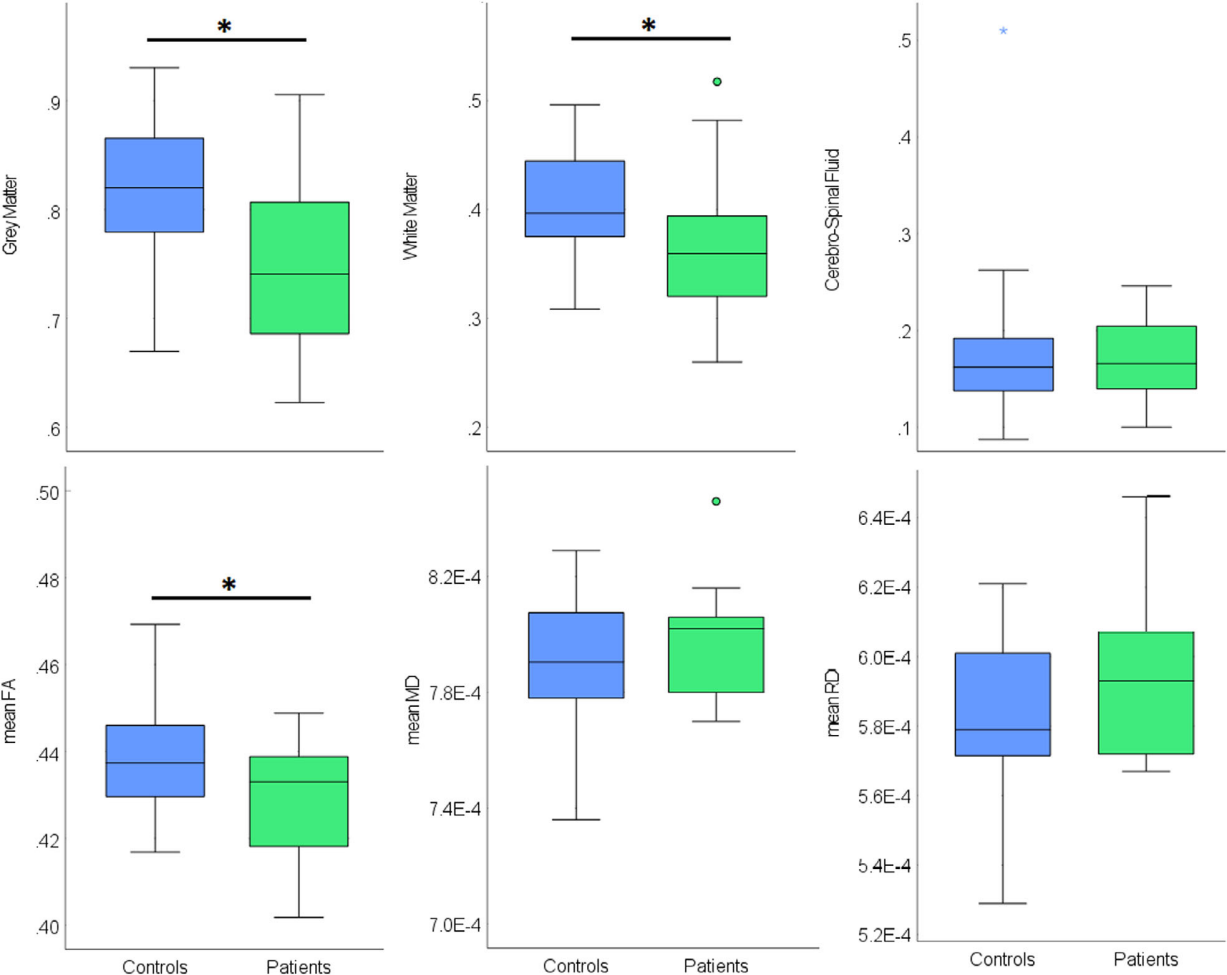
Group differences in whole brain measurements. Top: Grey matter, white matter and cerebrospinal fluid volumes (litre); Bottom panel: mean fractional anisotropy (FA), medial diffusivity (MD) and radial diffusivity (RD); in the control group (blue) and the patient group (green). Stars represent outliers, defined as values which are > 3 times the IQR either above Q3 or below Q1. Full circles represent values which are > 1.5 times the IQR. Black asterisk indicates significant group difference.

Whole brain measurements in each group can be seen in Figure 3. A 2 × 3 MANCOVA of whole brain tissue type volume revealed a significant main effect of group (*F*_(1,48)_ = 10.80, *p* = 0.002, partial eta squared = 0.184), and significant interaction between group and tissue type volume (*F*_(2,48) _= 4.08, *p* = 0.033, partial eta squared = 0.078). Post‐hoc tests showed that patients had lower GM and WM volumes than controls (*t*_(50)_ = 3.14, *p* = 0.002; *t*_(50)_ = 2.61, *p* = 0.006; for GM and WM respectively), but there was no group difference in CSF volume (*t*_(50) _= 0.59, *p* = 0.277).

Similarly, a 2 × 3 MANCOVA of whole brain diffusion parameters revealed a significant main effect of group (*F*_(1,41) _= 7.08, *p* = 0.011, partial eta squared = 0.147), and significant interaction between group and diffusion parameter (*F*_(2,41)_ = 7.06, *p* = 0.011, partial eta squared = 0.147). Post‐hoc tests showed that patients had significantly lower overall FA (*t*_(43)_ = 2.29, *p* = 0.014), but there was no difference in MD or RD between the groups (*t*_(43) _= 0.89, *p* = 0.189; *t*_(43) _= 1.41, *p* = 0.084; for MD and RD respectively).

Older age at CA, longer arrest time and having ECMO treatment were not associated with whole brain parameters (*p* > 0.05 for all). Table S6A provides statistics of the correlation analyses.

#### 3.2.2 Structures of interest

**FIGURE 4:**
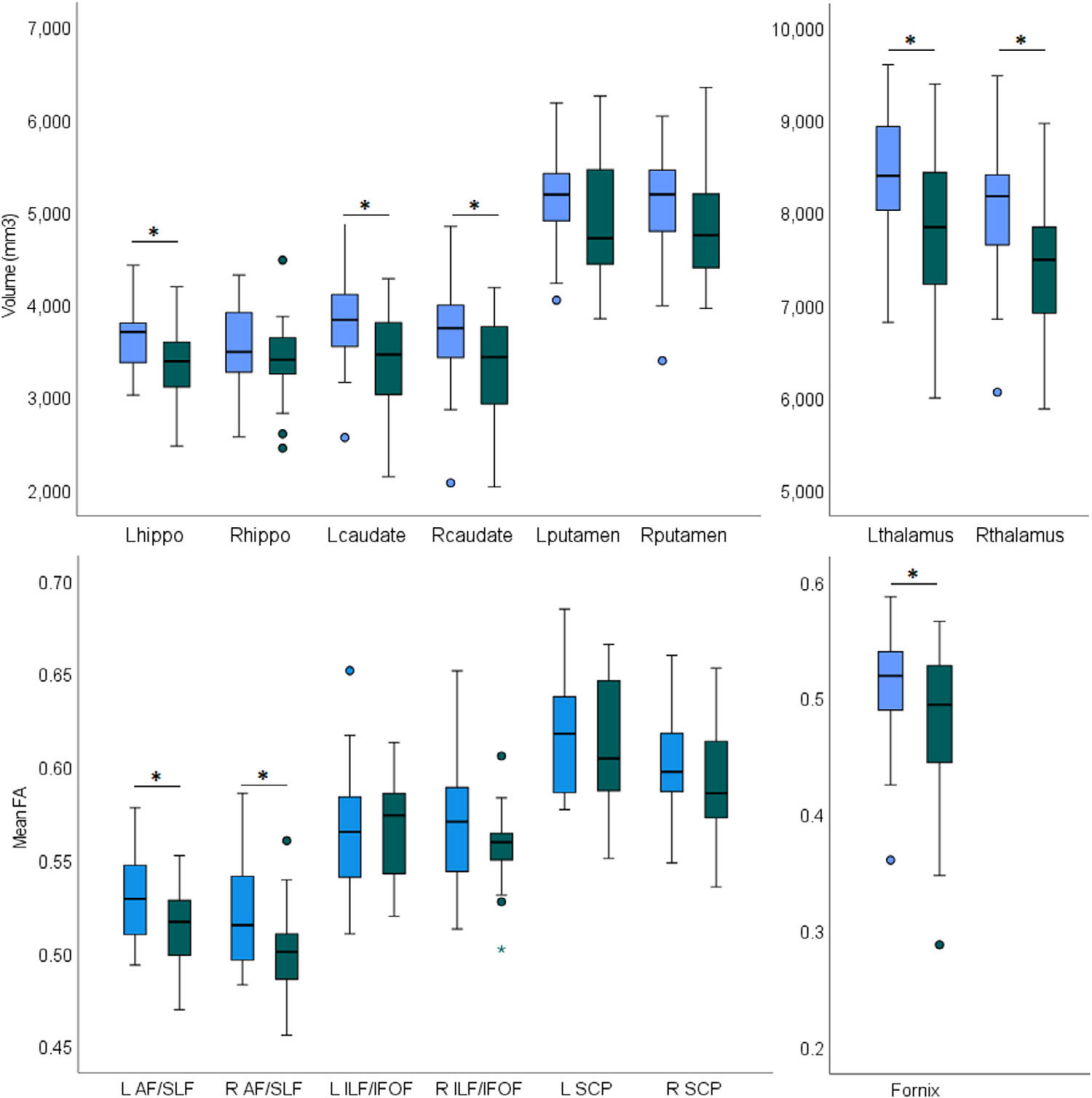
Group differences in structures of interest. Top: Volumes of the hippocampus (hippo), caudate nucleus (caudate), putamen and thalamus. Bottom: Mean FA of the AF/SLF, ILF/IFOF, SCP and fornix. Stars represent outliers, defined as values which are > 3 times the IQR either above Q3 or below Q1. Full circles represent values which are > 1.5 times the IQR. Black asterisk indicates significant group difference.

Having established that patients have lower whole brain white matter integrity (i.e., smaller volume and lower FA) and lower whole brain grey matter volume, we examined structures of interests. Group differences in structures of interest can be seen in Figure 4.

A 2 × 4 × 2 MANCOVA of subcortical structures volumes (hippocampus, thalamus, caudate nucleus and putamen) revealed a significant main effect of group (*F*_(1,47)_ = 10.16, *p* = 0.003, partial eta squared = 0.178), and significant interactions between structure and group (*F*_(3,47)_ = 4.23, *p* = 0.007, partial eta squared = 0.083), but not between hemisphere and group (*F*_(1,47)_ = 0.25, *p* = 0.619, partial eta squared = 0.005), and no significant 3‐way interaction (structure, hemisphere and group, *F*_(1,47)_ = 0.97, *p* = 0.408, partial eta squared = 0.020). Importantly, all significant effects disappeared when controlling for whole brain GM volume, suggesting that local changes are proportional to global GM reduction.

**FIGURE 5:**
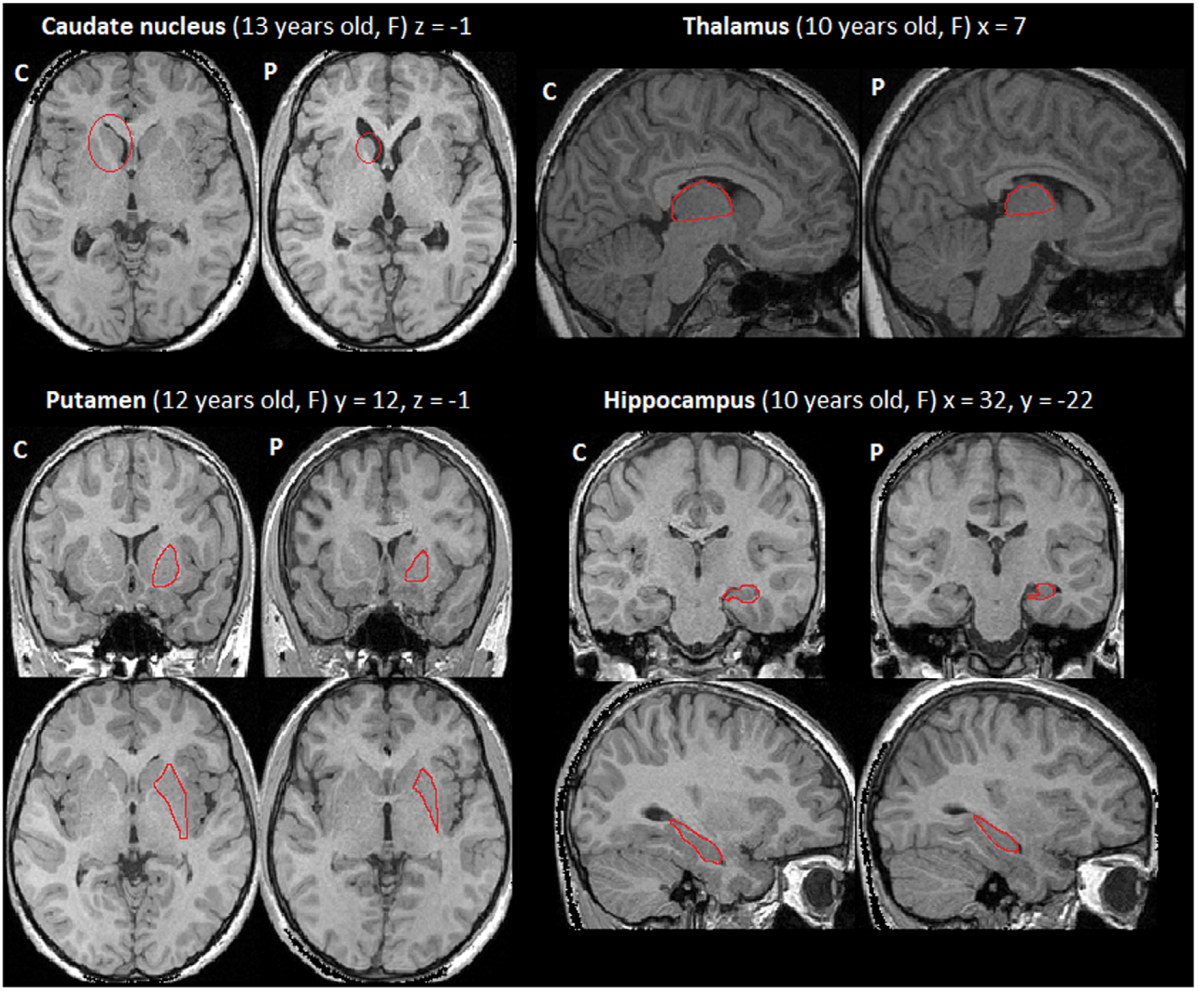
Examples of grey matter structures of interest in patient and control participants. T1‐weighted scans of a patient (P) and a control participant (C) matched for age and sex (in brackets). x, y, z coordinates are indicated above the images in MNI space. Brain structures are manually delineated or highlighted, for presentation purposes only.

Post‐hoc tests showed that patients had significantly reduced volume of the caudate nuclei and thalamus bilaterally (*t*_(49)_ = 3.14, *p* = 0.002; *t*_(49)_ = 2.54, *p* = 0.007; for left and right caudate; *t*_(49)_ = 2.66, *p* = 0.006; *t*_(49)_ = 2.86, *p* = 0.003; for left and right thalamus), and significantly smaller left hippocampus (*t*_(50)_ = 3.21, *p* = 0.001). The volume of the other subcortical structures did not significantly differ between patients and controls (*p* > 0.05 for all), nor did the volume of the brainstem, which acted as control structure (*p* > 0.05). Figure 5 presents the T1‐weighted scans of the patients with the smallest structure for each structure (as determined by the automatic segmentation), and the scan of a control participant most closely matched for age and sex.

In a 2 × 3 × 2 MANCOVA of FA in white matter tracts of interest (AF/SLF, ILF/IFOF and SCP), the interaction between hemisphere and group was significant (*F*_(1,41) _= 5.28, *p* = 0.027, partial eta squared = 0.114). All other effects were not significant (main effect of group, *F*_(1,41) _= 2.92, *p* = 0.095, partial eta squared = 0.066; interaction between tract and group, *F*_(2,40) _= 1.49, *p* = 0.234, partial eta squared = 0.035; 3‐way interaction between group, tract and hemisphere, *F*_(2,40) _= 2.78, *p* = 0.071, partial eta squared = 0.063). When controlling for global FA, the interaction between hemisphere and group remained significant. The significant effect was driven by patients having lower FA in bilateral AF/SLF (*t*_(43) _= 2.24, *p* = 0.015; *t*_(43) _= 2.20, *p* = 0.017, for right and left AF/SLF, respectively), with no group difference in the other tracts (*p* > 0.05 for all).

Lastly, patients’ fornix had significantly lower FA compared with the control group (*t*_(43) _= 1.87, *p* = 0.034), also when controlling for age, sex, and global FA.

### 3.3 Structure–function relationships

**FIGURE 6:**
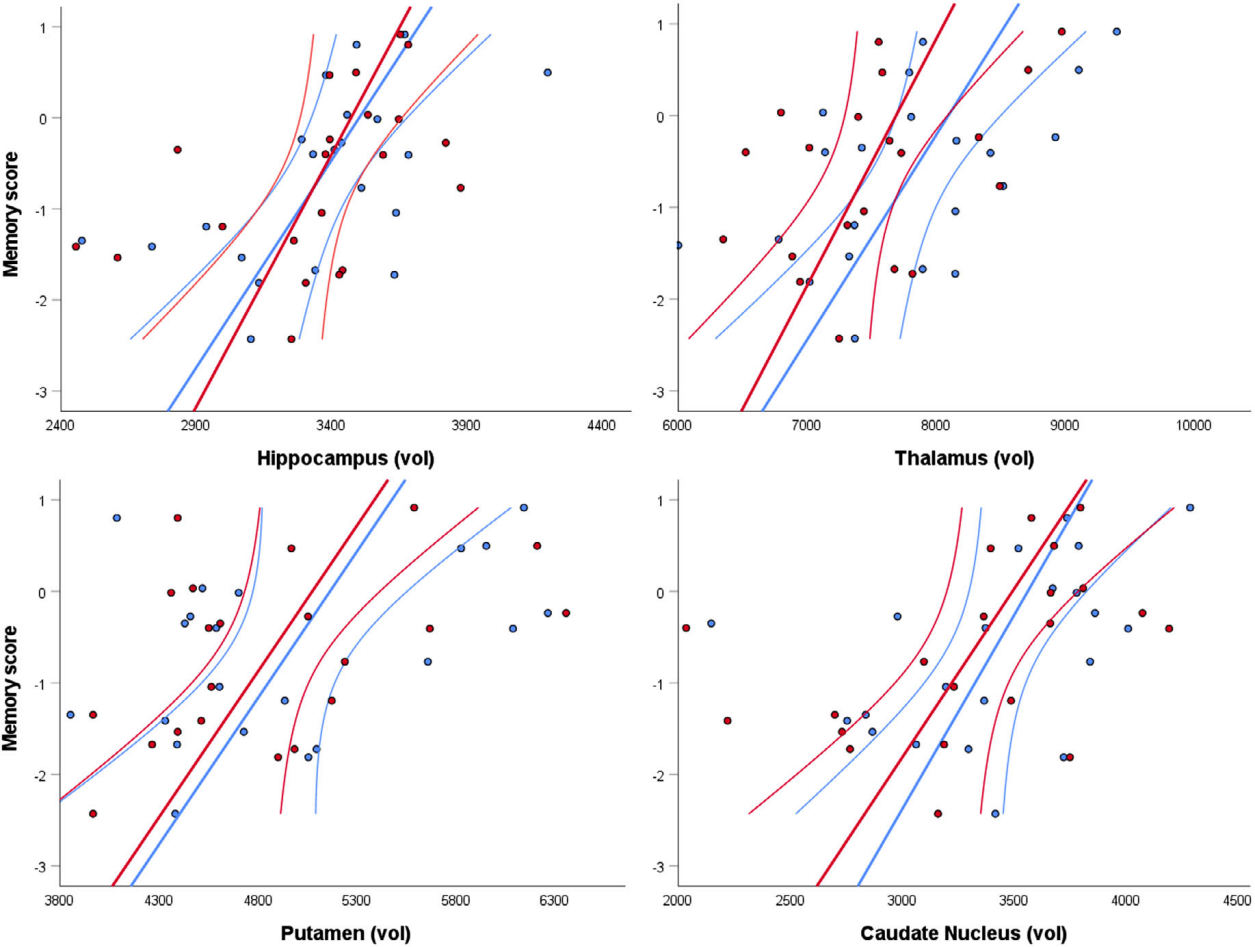
Correlations between memory scores and volumes of subcortical structures in the patient group. Y axis represents memory scores derived from the PCA; x axis represents volumes of right hemispheric (red) and left hemispheric (blue) subcortical structures (mm^3^); straight lines represent linear correlations; curved lines represent 95% confidence interval of the mean.

Within the patient group, better memory scores were associated with bilaterally larger volumes of the hippocampus (Left: Pearson's *r* = 0.55, *q* = 0.003; Right: *r* = 0.45, *q* = 0.03), thalamus (Left: *r* = 0.52, *q* = 0.03; Right: *r* = 0.45, *q* = 0.03), caudate nucleus (Left: *r* = 0.43, *q* = 0.04; Right: *r* = 0.44, *q* = 0.03) and putamen (Left: *r* = 0.47, *q* = 0.03; Right: *r* = 0.49, *q* = 0.03), but not with whole brain grey matter volume (*r* = 0.29, *p* = 0.102). See Figure 6.

Higher attainment scores were significantly associated with higher bilateral FA in the SCP (Left: *r* = 0.54, *q* = 0.049; Right: *r* = 0.61, *q* = 0.03), but not with global FA (*r* = 0.25, *p* = 0.163).

Language scores were not significantly correlated with any white or grey matter parameters (*q* > 0.05 for all non‐significant bivariate correlations) nor with whole brain grey matter volume or whole brain FA (*p* > 0.05).

Lastly, we note that adding global brain parameters as covariates to the calculation of partial correlations increases the p‐values of all correlations, with most remaining significant (Memory and Left hippocampus: Pearson's *r* = 0.49, *p* = 0.018; Right hippocampus: *r* = 0.39, *p* = 0.050; Left thalamus: *r* = 0.46, *p* = 0.024; Right putamen: *r* = 0.41, *p* = 0.042; Attainment scores and Left SCP: *r* = 0.51, *p* = 0.019; Right SCP: *r* = 0.58, *p* = 0.007), and others showing a trend (Memory and Right thalamus: *r* = 0.37, *p* = 0.063; Left caudate nucleus: *r* = 0.34, *p* = 0.076; Right caudate nucleus: *r* = 0.35, *p* = 0.074; Left putamen: *r* = 0.38, *q* = 0.055).

In contrast to the findings from the patient group, no significant structure‐function relationships were found in the group of control participants (*q* > 0.05 for all). Tables S6B and C provide statistics of the correlation analyses.

## 4 DISCUSSION

We studied a rare cohort of 28 children who suffered a cardiorespiratory arrest early in life, with the aim of establishing the behavioural and neuroanatomical sequalae of this HI event. Four main findings in the patient group define the functional and neuroanatomical outcome following paediatric CA: (i) The patient group had scores in the impaired range on memory, language and academic attainment; (ii) This was accompanied by lower values in measurements of global brain tissue volume and microstructure; (iii) Memory impairments were associated with bilaterally reduced volumes of subcortical grey matter structures, including the hippocampi, thalami, and striatum; and, (iv) Lower attainment scores were associated with reduced white matter FA in the superior cerebellar peduncle (SCP). We discuss each of these findings below.

### 4.1 Cognitive impairments and brain abnormalities

Previous studies of developmental outcome following HI have documented a range of cognitive deficits, including in the domains of memory (Cooper et al., 2015; Gadian et al., 2000; Muñoz‐López et al., 2017), language (Martinez et al., 2014; Robertson & Finer, 1985; Hövels‐Gürich et al., 2002), and attainment (Hövels‐Gürich et al., 2002; Marlow et al., 2005), though not necessarily simultaneously. Here we provide evidence that HI of cardiorespiratory origin can lead to impairments in all three domains. This finding is in line with a study of adult CA, which suggested that memory problems rarely appear in isolation, as even when studying only those patients who were referred to a memory clinic, mild to severe motor problems, and executive function deficits were found in all 10 patients who had memory impairments (Lim et al., 2004). We note that while as a group, the patients had a deficit in all three behavioural components, inter‐subject variability was large, and not all patients were impaired on all tasks (see Supplementary Tables 5A‐C). Specifically, as can be seen in Supplementary 2, Table 5C, some patients had preserved performance on all of the administered tests (e.g., P24), while others had impairments across multiple domains (e.g., P01, P23), and yet others had selective impairments (e.g., P04 having severe impairments only on tests of academic attainment, P11 with selective mild memory impairment, P16 and P19 have selective impairment in numerical operations, the former was later diagnosed with dyscalculia). This highlights the importance of administering a wide battery of tests, addressing cognitive function in various domains. It will be of interest in future studies to compare these individual profiles to those obtained from adults with a history of cardiac arrest. Still, based on the literature presented in the introduction, it seems that the variability of profiles mirrors, at least qualitatively, the behavioural profiles found in adults. In addition, variability between subtests was noted to be large as well, ranging from only one patient having impaired score on the MLU measurement, to third of patients showing impaired performance on the numerical operations subtest. Looking at the component scores, the language domain showed the lowest level of impaired performance, as only 36% of patients had a score within the impaired range (defined here as a score which is more than 1.5 standard deviations below the control group mean), followed by attainment, with 50% of patients having scores in the impaired range, and as hypothesised, the largest proportion of impaired performance was seen in the memory domain (79%). This was a consequence of the number of subtests in which the patient group had impaired performance (most of the subtests loading onto the Memory component, but only one subtest loading onto the Language component). We defined impaired performance on standardised tests according to clinical classification (on an individual level), and in comparison to the population mean (on a group level). Noticeably, for some ERRNI and WIAT subtests, the patient group performed significantly lower than the control group, but not significantly lower than the population mean. This reflects a common difficulty in cognitive studies, where the recruited control group performs better than expected from a completely random sample taken from the general population (Ganguli et al., 1998; Siritzky et al., 2023).

The patient group studied here also showed global brain abnormalities, which were driven by lower whole brain white and grey matter volumes (but not CSF), and lower whole brain FA (but not MD or RD). Whole brain white matter abnormalities have been documented previously in paediatric populations with history of HI. For example, lower white matter volumes were found in neonates treated for acute respiratory failure (Cooper et al., 2015), and children born prematurely (Soria‐Pastor et al., 2008; Northam et al., 2011). Widespread white matter abnormalities, quantified using diffusivity parameters (Mullen et al., 2011, Thompson, Lee et al., 2014), and VBM (Soria‐Pastor et al., 2008) have been documented in children born prematurely.

Looking at specific grey and white matter structures, we found that in our patient group, structures which are known to be vulnerable to HI in various paediatric populations, were affected bilaterally. This included reduced volumes of the hippocampus (Cooper et al., 2015; Isaacs et al., 2003; Muñoz‐López et al., 2017; Singh et al., 2019), thalamus (Dzieciol et al., 2017; Gadian et al., 2000; Singh et al., 2019), and caudate nucleus (Guderian et al., 2015; Singh et al., 2019). However, in accordance with the global reduction in grey matter volume in the patient group, the volume reduction in these specific grey matter structures was proportional to the reduction in whole brain grey matter volume. We also documented lower FA values in the bilateral AF/SLF (Mullen et al., 2011), and the fornix (Singh et al., 2019), which were not affected by controlling for global FA. Both mouse (Stone et al., 2008) and rat models (Delcour et al., 2012) of neonatal HI showed damage to the hippocampus and fornix, with the mouse model also providing evidence that the hippocampal damage precedes the degeneration of the fornix (Stone et al., 2008). As such, our findings are in line with these previous studies, confirming that the pattern of brain damage seen following CA is similar to that seen following other HI aetiologies.

### 4.2 Memory impairment and its neural correlates

In line with numerous previous studies, showing that hypoxic‐ischaemic damage can cause memory impairments in both the developing (Cooper et al., 2015; Gadian et al., 1999) and adult brain (Alexander et al., 2011; Bunch et al., 2004; Cronberg et al., 2009; Grubb et al., 1996; Mateen et al., 2011; Moulaert et al., 2009; O'Reilly et al., 2003; Sauve, 1995; Sunnerhagen et al., 1996; Tiainen et al., 2007), our study demonstrated that children who suffered CA early in life suffer from memory impairments. Within the patient group, memory impairments were more frequent than impairments in other domains, and were more frequent in the verbal domain, compared to visual domain.

It is also well established that the hippocampus, thalamus and dorsal striatum are especially vulnerable to HI (Fatemi et al., 2009), and in our study, reduced volume of these structures, but not of whole brain grey matter volume, was associated with lower performance on memory tasks. Below we discuss models and experimental findings on the role of each of these three structures in memory function, starting with the hippocampal formation.

Early models distinguished a ‘*memory formation’* from a ‘*habit formation’* system. The memory formation system was thought to be dependent on the cortical‐limbic‐thalamic circuit with the hippocampus as an essential hub, while the habit formation system was associated with the cortico‐striatal circuit (Mishkin et al., 1984). Below, we discuss each of these circuits and its potential relation to mnemonic processes, starting with the hippocampus‐based circuit. The involvement of the hippocampus in cognitive memory is now widely accepted, and early studies confirmed the relationship between early hypoxia‐induced hippocampal damage and selective memory disturbance (Cooper et al., 2015; Mishkin et al., 1998; Muñoz‐López et al., 2017; Vargha‐Khadem et al., 1997). Similarly, a study of patients with developmental amnesia following various HI events early in life, has shown that while patients have smaller than normal mid‐anterior and posterior thalamus, only the volume of the former correlated with memory function (Dzieciol et al., 2017). Based on studies of patients with thalamic damage who suffer memory impairments, and studies of animal models, it has been suggested that in adults, the anterior thalamic nuclei are a key node for integration of information (Aggleton & Brown, 1999; Hwang et al., 2017; Nelson, 2021; Sweeney‐Reed et al., 2021), a process which supports episodic memory formation (Aggleton, 2014). The mnemonic function of the anterior thalamic nuclei is therefore related to their structural connections to the hippocampal formation and the mammillary bodies (Grodd et al., 2020; reviewed in Sweeney‐Reed et al., 2021), but also to anatomical connections beyond the hippocampal circuity (Aggleton, 2014; Nelson, 2021).

There is also evidence that the striatum and its circuity are involved in various forms of learning and habit formation (Mishkin et al., 1984; Haber, 2016). During procedural learning (also described as ‘implicit’ or ‘reflexive’) the dorsal striatum (the posterior putamen and body and tail of caudate nucleus) support the process of making a learnt response automatic (Ashby et al., 1998; Ashby & Ennis, 2006; Cohen & Frank, 2009; Ell et al., 2011; Waldschmidt & Ashby, 2011). During rule‐based learning the dorsal striatum holds relevant, and filters out irrelevant information in the initial learning stages (Ashby & Ennis, 2006; Ell et al., 2006; Kovacs et al., 2021). In parallel, the hippocampus keeps track of accepted and rejected rules (Kovacs et al., 2021).

Further discussion of the role of the different dorsal striatal structures in learning is beyond the scope of the current study. However, we note that (i) it is widely accepted that the dorsal striatum is involved in various types of learning, beyond mere motor learning (Haber, 2016; Janacsek et al., 2022), supporting the findings from the current study, but (ii) only few studies have focused on the interplay between the hippocampal‐based circuit and the striatal‐based circuit(s) during learning processes, suggesting that, in adults, the hippocampus and caudate nucleus work together to consolidate learning [temporal (van de Ven et al., 2020) or spatial (Woolley et al., 2015)], therefore supporting memory formation.

In our study, lower integrity of both the striatum and the hippocampus was associated with compromised performance on complex memory tasks. Such performance can reflect difficulty in different stages and types of learning. Further disentanglement of the role of the caudate nucleus (head, body or tail), putamen (posterior or anterior) and hippocampus (with its multiple divisions) in learning and memory formation, will require future studies to employ (i) fine‐grained behavioural experimental paradigms, and; (ii) high resolution neuroimaging, in similar developmental populations to the one studied here.

### 4.3 Impairment in academic attainment and its neural correlates

A group level impairment on academic attainment was documented in our study. This score was based on a number of WIAT subtests. As the WIAT is a widely used standardised test, suitable for identifying children's strengths and weaknesses across a range of academic areas (Smith, 2001), it is an important tool in a cohort study like ours. However, performance on each of the subtests in this battery is dependent on multiple cognitive processes, and as a result, it is difficult to determine which cognitive process primarily contributed to the documented reduced performance and to the statistical association between task performance and the integrity of the cerebellar white matter tracts. Moreover, here we calculated an attainment factor, which included a few WIAT subtests. Looking at individual subtests, word reading and spelling loaded high on this factor (> 0.90) and numerical operations had slightly lower, though still substantial, factor loading (0.85). At the same time, patients were most impaired on the numerical operations subtest, with the group average significantly below the standard mean of 100, producing a large effect size. This parallels the findings of a recent study, showing that children who underwent heart transplant were impaired on the three WIAT subtests used here, with numerical operations producing the lowest group mean (Gold et al., 2020). While numerical operations are not often studied in developmental populations with history of HI, our study still replicates findings from some studies where difficulties with numerical operations were found (Isaacs et al., 2001; Taylor et al., 2011). We also note that Reading Comprehension was least sensitive to impairments in the patient group. Although we planned to include this subtest in our analyses, it was not included in the PCA as many participants did not complete it, due to time constraints.

In addition, we found that attainment scores were associated with the integrity of the SCP bilaterally, which is the main efferent white matter bundle of the cerebellum, but not with whole brain mean FA. Previous studies of children with cerebellar tumour resections have associated low SCP integrity [measured either using lesion symptom mapping (Albazron et al., 2019; Grosse et al., 2021); or FA (Law et al., 2011, 2017; Rueckriegel et al., 2015)] with general cognitive impairment (Albazron et al., 2019; Grosse et al., 2021; Rueckriegel et al., 2015), or more specifically, with impairments of working memory (Law et al., 2011, 2017). Together, these studies provide growing evidence for the relation between damage to the cerebellar efferent pathways and cognitive function in paediatric populations. Our study is the first to demonstrate this relation in a population with history of HI.

### 4.4 Impairments in language processing

The patient group in our study had impaired scores on the language component. At the same time, we also note that the group impairment was subtle, with most patients performing within the normal range (see Figure 1 and Supplementary Table 5). In addition, we did not find an association between any of the grey or white matter structures studied here, and the language scores. This might be a consequence of the aggregated nature of the score, i.e., it combines measurements of language comprehension and production, which are likely to depend on partially different neural network (Giglio et al., 2022). In contrast to our study results, previous reports of developmental outcome following neonatal HI have successfully associated the occurrence and severity of speech and language disorders with the level of damage to the basal ganglia (Martinez‐Biarge et al., 2010; Martinez‐Biarge, Bregant et al., 2012; Martinez‐Biarge, Diez‐Sebastian et al., 2012). In another study, verbal IQ was associated with watershed white matter damage (Steinman et al., 2009). In these previous studies patients had severe deficits and substantial damage which could be seen on clinical MRI images. In our study, severe visible damage was documented in only a handful of patients (see Supplementary Table 3) and cognitive impairments were much more subtle, which together might explain the difficulty in associating neural mechanisms and behavioural outcome. Future studies are needed to measure more fine‐grained cognitive mechanisms involved in language processing, which in turn might be more easily related to underlying neural mechanisms.

### 4.5 Study caveats and future work

Studying such a unique cohort of patients poses many challenges. First, we did not identify medical variables that could explain patients’ cognitive outcome. One possible explanation is that the variables recorded in this study were not sensitive enough to capture the variability in severity of the HI event. In line with our study, some previous investigations of adult populations have also failed to identify medical variables that predict functional outcome (Mateen et al., 2011; Moulaert et al., 2009; O'Reilly et al., 2003; Sunnerhagen et al., 1996; Tiainen et al., 2007). However, a study of neurodevelopmental status following neonatal TGA operation found that severe preoperative acidosis and hypoxia were associated with poor cognitive outcome (Hövels‐Gürich et al., 2002). Notably, this was defined based on more subtle measurements compared to those obtained in our study (e.g., pH value < 7.2 in umbilical venous blood), which in turn might contribute to better outcome prediction.

Second, individuals in our patient cohort suffered CA as a result of varied aetiologies, had various comorbidities (whether direct outcome from the main diagnosis or not), and varied in the presence, absence, and type of ongoing medical problems. These factors were not addressed in our statistical analyses, as the variability made it impossible to code them in a way that would allow for quantitative analysis. We suggest that future studies look at the overall severity of the medical condition, as defined by the combination of a few theoretically relevant medical variables, distinguishing those factors that lead to the CA and those that are present after the event but potentially affect the child's further development. Such aggregated score might be informative when it comes to predicting patients’ outcome.

Third, here we focused on subcortical grey matter structures and white matter tracts due to their known vulnerability to HI damage. However, it might be the case that a wider network is affected in our cohort as a result of the CA itself, later and/or ongoing medical issues, or brain reorganisation during development, and this in turn might further explain the behavioural variability in our cohort. Such networks have recently been delineated using resting state fMRI data (Barnett et al., 2021), and a study of a paediatric cohort with brain tumours demonstrated that altered connectivity in these networks was associated with CMS‐derived scores of verbal learning and recall (Alonso et al., 2023).

Fourth, the control group performed above the population mean in several standardised tests, which might explain some of the behavioural and anatomical group differences reported here. This is a result of a well‐documented self‐selection bias in psychological studies (Ganguli et al., 1998; Siritzky et al., 2023). To account for this bias, we (1) Compared the performance of the patient group to the population mean on all standardised tests; (2) Defined impaired performance on an individual level based on clinical standards; (3) Calculated correlations within group; and, (4) Recruited control participants from the full range of socio‐economic status (i.e., the control group did not differ from the patient group, nor from the general population, in SES). This is important as both cognitive function (Nisbett et al., 2012; Thomas & Coecke, 2023), and brain development (Thomas & Coecke, 2023) are known to be highly affected by SES. Still, future studies should attempt to select a control group which is representative of the general population in both SES and performance on cognitive tests.

Lastly, future studies should evaluate the implications of the behavioural deficits documented here on the quality of life of the children and/or their carers. This is especially important in light of previous findings in adults, showing that memory and other cognitive impairments resulting from hypoxic brain damage affect independence of patients, and the quality of life of both patients and their carers (Pusswald et al., 2000).

## 5 CONCLUSIONS

Children with a history of early cardiorespiratory arrest are an under‐studied group of patients. In this cohort study we have found that later in life, a substantial number of those children show deficits in the areas of memory, language and academic attainment. These domains require further investigation and should be addressed clinically in children with a similar medical history as the ones investigated here. Impairments in memory were highlighted in the past extensively, and currently it is well established that hypoxia‐ischaemia can lead to such outcome, in both adults and children. However, the deficit in language and academic attainment require further attention. As such, our findings of cognitive impairments across various domains, together with global brain abnormalities which are driven by damage to specific grey and white matter structures, add to the current understanding of long‐term outcome following paediatric HI.

## FUNDING STATEMENT

This work was supported by the Medical Research Council [grant numbers G0300117‐65439, G1002276‐98624]; and the UK Clinical Research Network. For the purpose of open access, the author has applied a Creative Commons Attribution (CC BY) licence to any Author Accepted Manuscript version arising.

## CONFLICT OF INTEREST DISCLOSURE

The authors declare no conflicts of interest.

## ETHICS APPROVAL STATEMENT

The study was approved by the London‐Bentham Research Ethics Committee (REC Reference No. 05/Q0502/88) and all participants and their carers read an age‐appropriate information sheet and gave written informed consent according to the Declaration of Helsinki, before commencing the study.

## Supporting information

Supporting information

Supporting information

## Data Availability

Data can be made available upon reasonable request from the corresponding author.

## ENDNOTE

1 Other medical variables previously shown to predict cognitive outcome following CA could not be examined statistically in our cohort. For example, whether patients had CA in‐ or out‐of‐hospital, or received Therapeutic Hypothermia.
