# Supplementary material for: Cognitive outcome and its neural correlates after cardiorespiratory arrest in childhood
Source: Dev Sci. 2024 Apr 1;27(4):e13501. doi: 10.1111/desc.13501 (PMC11753495; doi:10.1111/desc.13501)
Supplement: Supplementary file 1 — Supporting information [file DESC-27-e13501-s001.docx]

# Supplementary Material

## Figure 1. Inclusion and exclusion of patients from the cardiac arrest database.

Total (n=327)

Deceased (n=182)

Survivors (n=145)

Excluded (n=91)

**Medical** (n=55)

Genetic syndromes (n=19)

preterm birth (n=17)

Low birth weight (n=3)

Metabolic disorder (n=1)

Comorbid neuro conditions (n=15)

CVA pre-CA (n=5)

Brain tumour (n=1)

Meningitis (n=2)

Agenesis of the corpus callosum (n=1)

Epilepsy (n=3)

Cognitive delay prior to CA (n=2)

Multiple neuro events (n=1)

**Other** (n=36)

Lives outside the UK (n=25)

Parents are not English speakers

(n=3)

MR compatibility (n=7)

Social issues (n=1)

**Participants** n=28

Behavioural protocol; n=28

MRI scan; n=24

**Non-participants**

Refused (n=14)

Missing contact details (n=11)

No show (n=1)

Included (n=54)

## Table 1. Demographic and clinical information of individual participating patients.

| **ID** | **Age at test (years)** | **Sex** | **Primary aetiology** |  | **Diagnosis** | **CA age (years)** | **CA time (min)** | **ECMO duration (days)** |  |
| --- | --- | --- | --- | --- | --- | --- | --- | --- | --- |
| P01 | 12.3 | M | Respiratory failure |  | Interstitial lung disease, PH | 3.88 | 7 | 0 |  |
| P02 | 9.0 | M | Congenital Heart Disease |  | HLHS | 0.02 | 2 | 0 |  |
| P03 | 18.0 | F | Sepsis |  | Septic arthritis  ARDS | 13.78 | 1 | 8 |  |
| P04 | 14.2 | F | Congenital Heart Disease |  | AVSD | 4.33 | 5 | 0 |  |
| P05 | 9.4 | F | Congenital Heart Disease |  | TGA | 0.02 | 3 | 5 |  |
| P06 | 14.3 | M | Congenital Heart Disease |  | CoA, VSD | 0.02 | 7 | 0 |  |
| P07 | 11.2 | F | Congenital Heart Disease |  | ASD, PH | 10.37 | 7 | 0 |  |
| P08 | 15.2 | F | Acquired heart disease |  | Kawasaki disease  End-stage heart failure | 13.49 | 23† | 4 |  |
| P09 | 8.3 | F | Cardiomyopathy |  | DCM, End-stage heart failure | 0.73 | 4 | 10 |  |
| P10 | 10.7 | F | Arrhythmia |  | SVT | 0.05 | 3 | 0 |  |
| P11 | 10.5 | M | Congenital Heart Disease |  | CoA | 0.03 | 21† | 0 |  |
| P12 | 12.3 | F | Cardiomyopathy |  | DCM, End-stage heart failure | 13.38 | 100 | 10 |  |
| P13 | 9.0 | M | Respiratory failure |  | ARDS | 4.92 | 2 | 11 |  |
| P14 | 18.0 | F | Congenital Heart Disease |  | VSD, ASD, CoA | 1.44 | 3 | 7 |  |
| P15 | 14.2 | F | Sepsis |  | Sepsis | 6.36 | 2 | 0 |  |
| P16 | 9.4 | M | Cardiomyopathy |  | DCM, End-stage heart failure | 1.79 | 6 | 9 |  |
| P17 | 14.3 | M | Congenital Heart Disease |  | PA, VSD, PDA | 4.07 | 23 | 0 |  |
| P18 | 11.2 | M | Congenital Heart Disease |  | TAPVD | 0.04 | 12 | 6 |  |
| P19 | 15.2 | F | Congenital Heart Disease |  | Left atrial isomerism, double outlet right ventricle, congenital heart block, End-stage heart failure | 6.79 | 4 | 0 |  |
| P20 | 8.3 | M | Primary arrhythmia |  | Primary atrial multifocal arrhythmia, ASD | 0.22 | 7 | 19 |  |
| P21 | 10.7 | M | Congenital Heart Disease |  | TGA, VSD, pulmonary stenosis | 2.19 | 43 | 6 |  |
| P23 | 10.5 | F | Congenital Heart Disease |  | HLHS | 3.56 | 3 | 0 |  |
| P24 | 15.8 | M | Primary arrhythmia |  | Wolff Parkinson white syndrome | 0.04 | 4 | 0 |  |
| P25 | 8.3 | F | Congenital Heart Disease |  | TGA | 0.02 | 0.5 | 0 |  |
| P26 | 13.2 | F | Respiratory failure |  | Fetomaternal Transfusion | 0.00 | 1 | 0 |  |
| P27 | 13.3 | F | Respiratory failure |  | Pneumococcal bacterial infection  ARDS | 0.82 | 3 | 12 |  |
| P28 | 14.4 | F | Respiratory failure |  | Pneumococcal bacterial infection  ARDS | 2.92 | 4 | 14 |  |
| P29 | 12.5 | F | Respiratory failure |  | Meconium aspiration at birth | 0.00 | 4 | 5 |  |

† First CA occurred outside hospital. Abbreviations: ARDS = Acute respiratory distress syndrome; ASD = Atrial septal defect; AVSD = Atrioventricular septal defect; CA = cardiorespiratory arrest; CoA = Coarctation of the Aorta; DCM = Dilated Cardiomyopathy; ECMO = Extracorporeal membrane oxygenation machine; HLHS = Hypoplastic left heart syndrome; PA = Pulmonary Atresia; PDA = Patent Ductus Arteriosus; PH = Pulmonary hypertension; TAPVD = Total Anomalous Pulmonary Venous Drainage; TGA = Transposition of the great arteries; VSD = Ventricular Septal Defect.

## Table 2. Comparison of participating and non-participating patients

| **Variable** | | **Non-participants** | | **Participating Patients** | **Statistical test** | **p-value** |
| --- | --- | --- | --- | --- | --- | --- |
| **Sex** | Female | 14 | | 17 | Chi square | 0.610 |
|  | Male | 12 | | 11 |  |  |
| **Age at test (years)** † | Mean | 11.46 | | 12.04 | t-test | 0.550 |
|  | Std. Deviation | 3.67 | | 3.34 |  |  |
|  | Range | 7 – 21 | | 8 – 20 |  |  |
| **SES** | Mean | 14,614 | | 17,241 | Mann-Whitney | 0.346 |
|  | Std. Deviation | 10,700 | | 9,367 |  |  |
| **CA Location** | In Hospital | 26 | | 27 | Chi square | 0.331 |
|  | Outside Hospital | 0 | | 1 |  |  |
| **Mechanical circulatory support** | No | 25 | | 27 | Chi square | 0.957 |
|  | Yes | 1 | | 1 |  |  |
| **Heart Transplant** | No | 21 | | 22 | Chi square | 0.841 |
|  | Yes | 5 | | 6 |  |  |
| **Number of CA** | 1 | 15 | | 21 | Chi square | 0.282 |
|  | 2 | 7 | | 3 |  |  |
|  | 3 | 4 | | 4 |  |  |
| **Age at first CA (days)** | Median | 333.5 | 589.0 | | Mann-Whitney | 0.703 |
|  | IQR | 1344 | 1729 | |  |  |
| **CA Total Time (min)** | Median | 5 | 4 | | Mann-Whitney | 0.531 |
|  | IQR | 11 | 4 | |  |  |
| **Days on ECMO** | Median | 0 | 2 | | Mann-Whitney | 0.269 |
|  | IQR | 5 | 9 | |  |  |

† For the non-participant group, age indicates the patient’s age on the day when we initially attempted to contact the caregivers for the purpose of study recruitment. Abbreviations: CA = cardiac arrest, ECMO = extracorporeal membrane oxygenation, IQR = interquartile range, SES = socio-economic status.

## Table 3. Radiological findings on MRI.

| **ID** | **Hippo** | **Fornix** | **MB** | **S > G** | **BG** | **Peri-ventricular** | **Lateral ventricle** | **Other** |
| --- | --- | --- | --- | --- | --- | --- | --- | --- |
| P02 | N | N | N | N | N | N | N | None |
| P03 | N | N | N | N | N | N | N | Old haematoma in genu of CC, R anterior limb of internal capsule and L parietal WM; possible gliotic scar in L deep frontal WM |
| P04 | N | N | N | N | N | N | N | None |
| P05 | N | N | N | N | N | N | N | Slightly small CC |
| P06 | Sm | Sm | Sm | N | N | Slightly reduced, Bilateral gliotic scars | Slightly large | None |
| P07 | N | N | N | N | N | N | N | Possible pituitary haemorrhage; possibly slightly small CC |
| P08 | N | N | N | N | N | N | N | None |
| P10 | N | N | N | N | N | N | N | None |
| P12 | N | N | N | N | N | N | N | None |
| P13 | N | N | N | N | N | N | N | None |
| P14 | N | N | Sm | N | R Caudate injury | Reduced | Dilated | R frontal cortical scar and deep WM gliotic scars |
| P15 | N | N | N | N | N | N | N | None; dental artefacts |
| P16 | N | N | N | N | N | N | N | None |
| P17 | N | N | N | N | N | N | N | None |
| P18 | N | N | N | N | N | N | N | None |
| P19 | N | N | N | S > G | R Caudate & Putamen gliotic scar | N | N | None |
| P20 | N | N | N | N | N | N | N | Small gliotic scar in right frontal WM |
| P21 | N | N | N | N | N | N | N | Bilateral parieto-occipital cortical injury and small optic chiasm and anterior visual pathways |
| P23 | N | N | N | N | N | N | N | None |
| P24 | N | N | N | N | N | N | N | None |
| P25 | N | N | N | N | N | N | N | None |
| P26 | N | Sm | Sm | N | N | N | N | R frontal cortical injury; small CC |
| P27 | N | N | N | N | N | N | N | None |
| P28 | N | N | N | N | L Caudate & Putamen gliotic scar | N | N | R frontal operculum and insula cortex injury; including post central gyrus |
| P29 | N | N | N | N | N | N | N | Slightly small CC |

The following pre-defined brain structures were rated as either normal or small on inspection: hippocampus, mesial-temporal lobe, fornix, dorsomedial thalamus, mammillary bodies, basal ganglia, and cerebellum. The size of the mammillary bodies was evaluated in relation to two internal landmarks: the optic chiasm and the anterior commissure. The lateral ventricle was rated for being normal or enlarged/dilated. Periventricular white matter was rated for amount (normal or reduced), and for presence of abnormalities such as scars. The size of the genu and the splenium of the corpus callosum was compared, where splenium > genu was rated as normal; while a reversed relationship (genu > splenium) was rated as abnormal. Other abnormalities such as ischaemic or atrophic damage, haematoma or gliotic scars were noted in 11 patients. The scans of all healthy controls were rated as normal. P01, P09, P11 had no MRI scan. The mesial temporal lobe, dorsomedial thalamus and cerebellum were rated as normal in all patients and are therefore not listed in this Table. Abbreviations: BG = Basal Ganglia; CC = corpus callosum; Hippo = hippocampus; L = left; MB = mammillary bodies; N = normal; R = right; S > G = Splenium > Genu; Sm = small; WM = white matter.

## Table 4. Factor Loading of Principal Component Analyses

| **PCA 1: Memory** | | **PCA 2: Language** | | **PCA 3: Attainment** | |
| --- | --- | --- | --- | --- | --- |
| *Variance* | 54.41% | *Variance* | 59.9% | *Variance* | 82.1% |
| *KMO* | 0.7 | *KMO* | 0.66 | *KMO* | 0.7 |
| *Bartlett’s χ^2^* | 41.5 | *Bartlett’s χ^2^* | 83.9 | *Bartlett’s χ^2^* | 95.8 |
| *Bartlett’s p* | < 0.001 | *Bartlett’s p* | < 0.001 | *Bartlett’s p* | < 0.001 |
| **Factor loading** | | | | | |
| Sunderland | -0.81 | Ideas 1 | 0.87 | Numerical operations | 0.85 |
| Verbal delayed | 0.85 | Ideas 2 | 0.91 | Word reading | 0.93 |
| RBMT | 0.81 | MLU | 0.51 | Spelling | 0.93 |
| ERRNI Forgetting | 0.39 | Comprehension | 0.74 |  |  |

ERRNI = The Expression, Reception and Recall of Narrative Instrument; KMO = Kaiser–Meyer–Olkin measure of adequacy; MLU = mean length of utterance; RBMT = Rivermead Behavioural Memory Test; WIAT = Wechsler Individual Achievement Test.

## Table 5. Behavioural scores and Results of Principal Component analyses

|  | **Control group** | | | **Patient group** | | |
| --- | --- | --- | --- | --- | --- | --- |
|  | **Mean** | **Standard Deviation** | **Impaired performance** | **Mean** | **Standard Deviation** | **Impaired performance** |
| Memory |  |  |  |  |  |  |
| Sunderland (Z score) | -0.55 | 0.45 | 0/25 | 0.81 | 1.55 | 0/26 |
| Verbal Delayed | 113.86 | 10.63 | 0/28 | 88.52 | 17.81 | 6/27 (22%) |
| RBMT (% correct) | 92.56 | 6.94 | 0/27 | 83.05 | 13.04 | 5/27 (19%) |
| ERRNI Forgetting | 101.74 | 11.48 | 1/27 (4%) | 97.11 | 12.14 | 2/28 (7%) |
| **Memory (PCA)** | **0.65** | **0.37** | **0/25** | **-0.68** | **1.00** | **19/24 (79%)** |
| Language (ERRNI) |  |  |  |  |  |  |
| Ideas 1 | 112.41 | 12.73 | 0/27 | 106.93 | 16.98 | 1/28 (3.5%) |
| Ideas 2 | 115.07 | 12.08 | 0/27 | 104.71 | 17.83 | 2/28 (7%) |
| MLU | 110.41 | 15.95 | 1/27 (4%) | 100.79 | 12.13 | 1/28 (3.5%) |
| Comprehension | 108.19 | 9.02 | 0/27 | 98.21 | 14.25 | 4/28 (14%) |
| **Language (PCA)** | **0.38** | **0.63** | **2/27 (8%)** | **-0.37** | **1.15** | **10/28 (36%)** |
| Attainment (WIAT) |  |  |  |  |  |  |
| Numerical Operations | 116.36 | 17.79 | 0/28 | 89.00 | 23.99 | 9/27 (33%) |
| Word Reading | 110.16 | 11.79 | 1/25 (4%) | 94.07 | 18.95 | 6/27 (22%) |
| Spelling | 107.20 | 13.71 | 1/25 (4%) | 93.04 | 15.84 | 6/26 (23%) |
| **Attainment (PCA)** | **0.54** | **0.67** | **2/25 (7%)** | **-0.51** | **1.00** | **13/26 (50%)** |
| Reading Comprehension | 116.17 | 8.38 | 0/18 | 99.27 | 14.01 | 2/26 (8%) |
| Additional tests |  |  |  |  |  |  |
| WASI VIQ | 116.54 | 11.74 | 0/28 | 99.25 | 16.61 | 4/28 (14%) |
| WASI PIQ | 109.54 | 11.20 | 0/28 | 94.50 | 13.43 | 3/28 (11%) |
| WASI FSIQ | 114.46 | 9.46 | 0/28 | 96.36 | 14.33 | 3/28 (11%) |
| Visual Immediate | 103.79 | 16.12 | 2/28 (7%) | 101.67 | 14.12 | 2/27 (7%) |
| Visual Delayed | 104.07 | 14.94 | 2/28 (7%) | 97.59 | 13.29 | 4/27 (15%) |
| Verbal Immediate | 113.96 | 13.27 | 0/28 | 93.07 | 17.30 | 6/27 (22%) |
| Delayed Recognition | 106.32 | 9.30 | 0/28 | 92.26 | 15.83 | 5/27 (19%) |

### 5A. Performance scores on the tests for the patient and the control groups.

Impaired performance [number (percentage) of participants/total n] is defined as follows: Sunderland, PCA components: a score which is more than 1.5 standard deviations below the mean; WIAT, ERRNI, CMS/WMS, WASI: scores below 80, which is low or exceptionally low score according to clinical classification; RBMT: impaired range according to manual.

### 5B. Group differences in behavioural scores

| **Test** | **Group difference**  **Patients vs Controls** | | | | **Group performance**  **Patients vs population mean (μ)** | | |
| --- | --- | --- | --- | --- | --- | --- | --- |
|  | **Stats** | **value** | **df** | **p** | **t** | **df** | **p** |
| **CMS/WMS** |  |  |  |  |  |  |  |
| Visual Immediate | F | 0.268 | 1,53 | 0.607 | 0.613 | 26 | 0.545 |
| Visual Delayed | F | 2.880 | 1,53 | 0.096 | -0.941 | 26 | 0.355 |
| Verbal Immediate | F | 25.359 | 1,53 | <0.001 | -2.080 | 26 | 0.048 |
| Verbal Delayed | F | 41.407 | 1,53 | <0.001 | -3.350 | 26 | 0.002 |
| Delayed Recognition | F | 16.272 | 1,53 | <0.001 | -2.541 | 26 | 0.017 |
| **Memory** |  |  |  |  |  |  |  |
| RBMT (% correct) | U | 3.251 | 51 | 0.001 | N/A |  |  |
| Sunderland (Z score) | U | 3.079 | 54 | 0.002 | 2.650 | 25 | 0.014 |
| **ERRNI** |  |  |  |  |  |  |  |
| Ideas 1 | F | 1.823 | 1,53 | 0.183 | 2.160 | 27 | 0.040 |
| Ideas 2 | F | 6.315 | 1,53 | 0.015 | 1.399 | 27 | 0.173 |
| MLU | F | 2.113 | 1,53 | 0.152 | 0.343 | 27 | 0.735 |
| Comprehension | F | 9.529 | 1,53 | 0.003 | -0.663 | 27 | 0.513 |
| Forgetting | F | 6.367 | 1,53 | 0.015 | -1.261 | 27 | 0.218 |
| **WIAT** |  |  |  |  |  |  |  |
| Numerical Operations | F | 23.197 | 1,53 | <0.001 | -2.383 | 26 | 0.025 |
| Word Reading | F | 13.252 | 1,50 | 0.001 | -1.625 | 26 | 0.116 |
| Spelling | F | 11.609 | 1,49 | 0.001 | -2.241 | 25 | 0.034 |
| Reading Comprehension | F | 20.898 | 1,42 | <0.001 | -0.266 | 25 | 0.792 |
| **WASI** |  |  |  |  |  |  |  |
| WASI VIQ | F | 20.230 | 1,53 | <0.001 | -0.239 | 27 | 0.813 |
| WASI PIQ | F | 20.707 | 1,53 | <0.001 | -2.168 | 27 | 0.039 |
| WASI FSIQ |  |  |  |  | -1.345 | 27 | 0.190 |

Behavioural performance was compared between the two participant groups (patients and control group) using one-way ANOVA (F reported) or Mann-Whitney U test (U reported). The performance of the patient group was also compared to the population mean (μ) on standardised tests (all tests other than the RBMT) using 1-sample, 2-tailed t-tests. WASI, CMDS/WMS, ERNNI and WIAT μ = 100, Sunderland μ = 0.

### 5C. Inter-subject variability in behavioural performance

The excel table presents coding of impairment level for each participant, and each task, for all standardised tests. Coding of the RBMT according to manual: Normal (Green), Poor memory / borderline (Orange), Impaired / moderately to severely impaired (Red). Coding for the WASI, CMS/WMS, WIAT and ERRNI according to UK clinical and educational standards: 0-79 - Extremely Low range to Borderline range (Red), 80-89 - Low/below Average range (Orange), > 90 - Average or above average range (Green). Missing data is indicated in Grey.

## Table 6. Correlation matrices

We report below partial correlations with age as covariate. P-values are adjusted using Benjamini–Hochberg FDR corrections for multiple comparisons. * indicates that p-value is significant at an uncorrected threshold of p < 0.05, ** indicates that adjusted p-value (q) is significant at threshold of p < 0.05. Abbreviations: Fractional Anisotropy (FA), Medial Diffusivity (MD), Radial Diffusivity (RD), Arcuate fasciculus/superior longitudinal fasciculus (AF/SLF), Inferior longitudinal fasciculus/Inferior fronto-occipital fascicle (ILF/IFOF), Right (R), Left (L).

### 6A. Correlations between medical variables and behavioural performance or whole brain measures

| **Variable** | **Statistic** | **Age at first cardiac arrest** | **Total Time of cardiac arrest** |
| --- | --- | --- | --- |
| Regression factor score for academic attainment | Correlation | 0.139 | -0.246 |
|  | P-value | 0.291 | 0.163 |
|  | p-value adjusted | 0.36 | 0.34 |
| Regression factor score for language | Correlation | 0.565 | 0.246 |
|  | P-value | 0.007 | 0.163 |
|  | p-value adjusted | 0.09 | 0.34 |
| Regression factor score for Memory | Correlation | 0.153 | -0.009 |
|  | P-value | 0.272 | 0.485 |
|  | p-value adjusted | 0.36 | 0.49 |
| Whole brain grey matter volume | Correlation | -0.535 | -0.216 |
|  | P-value | 0.011 | 0.194 |
|  | p-value adjusted | 0.09 | 0.34 |
| Whole brain white matter volume | Correlation | -0.117 | -0.089 |
|  | P-value | 0.322 | 0.363 |
|  | p-value adjusted | 0.37 | 0.39 |
| Mean whole brain FA | Correlation | 0.298 | -0.239 |
|  | P-value | 0.114 | 0.169 |
|  | p-value adjusted | 0.34 | 0.34 |
| Mean whole brain MD | Correlation | -0.363 | 0.152 |
|  | P-value | 0.070 | 0.274 |
|  | p-value adjusted | 0.31 | 0.36 |
| Mean whole brain RD | Correlation | -0.353 | 0.203 |
|  | P-value | 0.076 | 0.210 |
|  | p-value adjusted | 0.31 | 0.34 |

### 6B. Correlations between behavioural performance and white matter tracts integrity

| **Variables** | **Statistics** | **Regression factor score for academic attainment** | **Regression factor score for language** | **Regression factor score for memory** |
| --- | --- | --- | --- | --- |
| R AF/SLF FA | Correlation | 0.158 | 0.361 | 0.263 |
|  | P-value | 0.265 | 0.071 | 0.146 |
|  | p-value adjusted | 0.35 | 0.22 | 0.33 |
| L AF/SLF FA | Correlation | 0.069 | 0.357 | 0.192 |
|  | P-value | 0.393 | 0.073 | 0.222 |
|  | p-value adjusted | 0.40 | 0.22 | 0.34 |
| R ILF/IFOF FA | Correlation | 0.162 | 0.162 | 0.316 |
|  | P-value | 0.260 | 0.260 | 0.101 |
|  | p-value adjusted | 0.35 | 0.5 | 0.33 |
| L ILF/IFOF FA | Correlation | 0.143 | 0.002 | 0.022 |
|  | P-value | 0.286 | 0.497 | 0.465 |
|  | p-value adjusted | 0.35 | 0.5 | 0.48 |
| L Superior Cerebellar Peduncle FA | Correlation | 0.535 | -0.028 | 0.344 |
|  | P-value | 0.011** | 0.456 | 0.081 |
|  | p-value adjusted | 0.049 | 0.5 | 0.33 |
| R Superior Cerebellar Peduncle FA | Correlation | 0.612 | -0.015 | 0.278 |
|  | P-value | 0.003** | 0.477 | 0.132 |
|  | p-value adjusted | 0.03 | 0.5 | 0.33 |
| Fornix FA | Correlation | -0.280 | 0.362 | 0.189 |
|  | P-value | 0.130 | 0.070 | 0.226 |
|  | p-value adjusted | 0.35 | 0.22 | 0.34 |
| **Mean whole brain FA^§^** | Correlation | 0.246 | -0.063 | 0.256 |
|  | P-value | 0.163 | 0.401 | 0.153 |

**^§^** Correlation between performance and Mean whole brain FA acts as a control, and therefore adjusted p-value is not reported, demonstrating that even at a lenient uncorrected p-value threshold, correlation was not significant.

### 6C. Correlations between behavioural performance and grey matter structures volume

| **Variables** | **Statistics** | **Regression factor score for academic attainment** | **Regression factor score for language** | **Regression factor score for memory** |
| --- | --- | --- | --- | --- |
| L hippocampus | Correlation | .329 | .141 | .550 |
|  | P-value | .078 | .277 | .006** |
|  | p-value adjusted | 0.29 | 0.32 | 0.03 |
| R hippocampus | Correlation | -.083 | .485 | .449 |
|  | P-value | .364 | .015* | .024** |
|  | p-value adjusted | 0.37 | 0.13 | 0.03 |
| L thalamus | Correlation | .223 | .093 | .518 |
|  | P-value | .173 | .348 | .010** |
|  | p-value adjusted | 0.29 | 0.35 | 0.03 |
| R thalamus | Correlation | .162 | .155 | .453 |
|  | P-value | .248 | .258 | .022** |
|  | p-value adjusted | 0.34 | 0.32 | 0.03 |
| L caudate nucleus | Correlation | .082 | .408 | .425 |
|  | P-value | .366 | .037* | .031** |
|  | p-value adjusted | 0.37 | 0.15 | 0.04 |
| R caudate nucleus | Correlation | .216 | .331 | .441 |
|  | P-value | .180 | .077 | .026** |
|  | p-value adjusted | 0.29 | 0.21 | 0.03 |
| L putamen | Correlation | .306 | .193 | .466 |
|  | P-value | .095 | .208 | .019** |
|  | p-value adjusted | 0.29 | 0.32 | 0.03 |
| R putamen | Correlation | .222 | .142 | .488 |
|  | P-value | .173 | .275 | .014** |
|  | p-value adjusted | 0.29 | 0.32 | 0.03 |
| **Whole brain grey matter volume** | Correlation | .255 | -.103 | .297 |
|  | P-value | .139 | .333 | .102 |

**^§^** Correlation between performance and whole brain grey matter volume acts as a control, and therefore adjusted p-value is not reported, demonstrating that even at a lenient uncorrected p-value threshold, correlation was not significant.
